# Endoscope-assisted versus conventional neck dissection in patients with oral cancer: a systematic review and meta-analysis

**DOI:** 10.1186/s40463-022-00567-9

**Published:** 2022-05-11

**Authors:** Yi-Chan Lee, Li-Jen Hsin, Shih-Wei Yang, Ming-Shao Tsai, Yao-Te Tsai, Che-Fang Ho

**Affiliations:** 1 Department of Otolaryngology - Head and Neck Surgery, Chang Gung Memorial Hospital, No. 222, Maijin Rd., Anle Dist., Keelung City, 204 Taiwan; 2 Department of Otolaryngology - Head and Neck Surgery, Chang Gung Memorial Hospital, Taoyuan, Taiwan; 3 Department of Otolaryngology—Head and Neck Surgery, Chang Gung Memorial Hospital, Chiayi, Taiwan; 4 College of Medicine, Chang Gung University, Taoyuan, Taiwan

**Keywords:** Endoscope, Neck dissection, Neck lymphadenectomy

## Abstract

**Background:**

Neck dissection is an integral component of the treatment of head and neck cancers. The present meta-analysis aimed to compare the use of endoscope-assisted neck dissection (END) with conventional neck dissection (CND) in the existing English literature.

**Methods:**

A search of PubMed (MEDLINE), Embase, and the Cochrane Library for articles reporting the results of the two techniques of neck dissection was completed independently by two individuals. The authors analyzed the data from each study using a random-effects model.

**Results:**

The pooled analysis demonstrated comparable lymph node yield, intraoperative blood loss, incidence of locoregional recurrence, and incidence of complications between the two groups. A significantly longer operative time but a shorter length of hospital stay was observed in the END group compared with the other group.

**Conclusions:**

Compared with conventional techniques, END offers similar oncologic outcomes and complication rates; however, it requires a longer operative time. Future studies with long-term follow-up and assessment of patient satisfaction are needed to confirm the clinical use of END.

**Graphical abstract:**

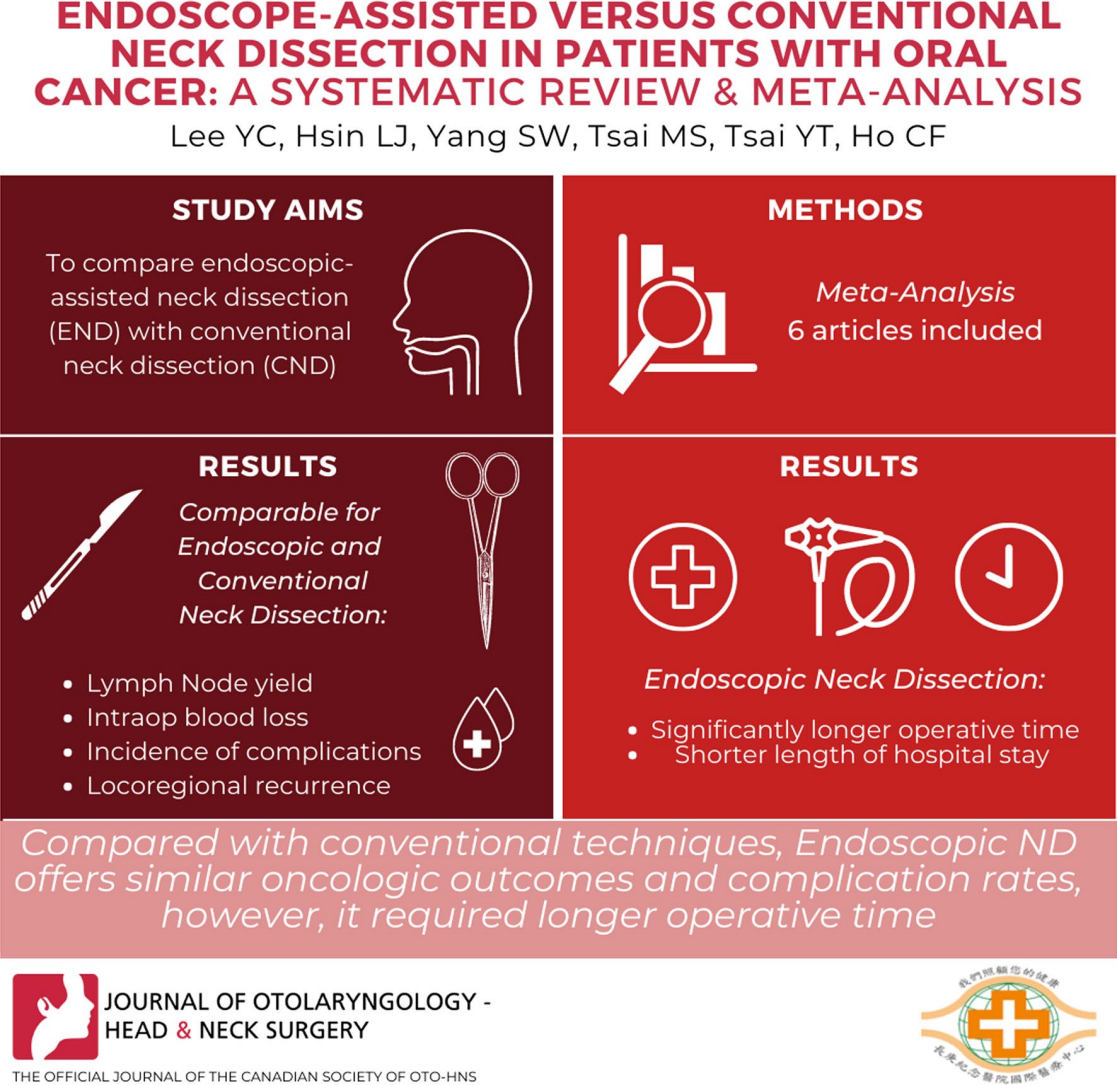

**Supplementary Information:**

The online version contains supplementary material available at 10.1186/s40463-022-00567-9.

## Background

Neck dissection (ND) plays a fundamental role in the treatment of head and neck cancers. The procedures outlined by Crile in 1906 described the standard form of ND, and several modifications were subsequently developed [1]. With advances in surgical equipment and techniques, physicians have been making progress in improving quality of life without compromising oncologic safety. The use of robots or endoscopes to assist surgical procedures may represent a potential method to achieve this goal [2].

The use of robotic systems in ND has been reported by several research groups [3, 4]. A meta-analysis has also been conducted to compare the differences between robotic and conventional neck dissection (CND) [5]. However, the cost of infrastructure, training and the learning curve represent challenges for robotic ND to be universally accessible. By contrast, endoscope-assisted surgeries represent an alternative approach in terms of cost-effectiveness. The present meta-analysis aimed to compare the use of endoscope-assisted neck dissection (END) with CND in the existing English literature.

## Methods

### Data sources and collection

The authors conducted this study on the basis of the Preferred Reporting Items for Systematic Reviews and Meta-Analyses (PRISMA) statement [6]. Two of the authors searched PubMed, Embase, and the Cochrane Library independently and extensively for articles of interest published before June 2021. The keywords used in the search process included “endoscopy”, “endoscope”, “neck lymphadenectomy”, “cervical lymphadenectomy”, “neck dissection” and “lymph node dissection”. Moreover, a comprehensive review of the reference lists of the included studies was performed to identify relevant articles.

### Study selection and data extraction

We included studies according to the following criteria: studies including oral cancer patients who received ND and studies reporting the results of ND between endoscope-assisted and conventional approaches. Studies not published in English, studies not including CND as a control group, animal studies, short reports and abstracts were excluded. Data of interest were collected by two authors independently. The authors evaluated the bias of the articles with the Newcastle–Ottawa Scale for nonrandomized studies and Cochrane Collaboration’s risk of bias tool (RoB 1.0) for randomized studies [7, 8]. Bias assessment differences were discussed among the authors until mutual agreement was reached.

### Outcomes

The main outcomes of this study were lymph node (LN) yield, operative time, intraoperative blood loss, length of hospital stay, locoregional recurrence and complication rate. Cosmetic satisfaction regarding scars was not reported by most included studies, making pooled analysis impossible.

### Data analyses

Statistical analyses were performed using Comprehensive Meta-Analysis software (version 3), Biostat, Englewood, NJ, USA. Mean differences (MDs) were used for the comparison of the total LN yield, operative time, amount of intraoperative blood loss and length of hospital stay between the END and CND groups. Risk differences (RDs) were used to compare the incidences of nodal recurrence and the incidence of postoperative complications between the two groups. If necessary, the mean and standard deviation were estimated using methods reported in previous studies [9, 10]. We used a random-effects model to perform all the analyses. The heterogeneity of the studies was calculated with the *I*^*2*^ statistic. The level of heterogeneity was considered low, medium and high if the *I*^*2*^ values were 25–50%, 50–75% and ≥ 75%, respectively [11]. The potential for publication bias was assessed by funnel plot analysis and Egger’s intercept tests [11]. Statistical significance was defined as a 2-tailed *P*-value of less than 0.05.

## Results

### Study selection

**Fig. 1:**
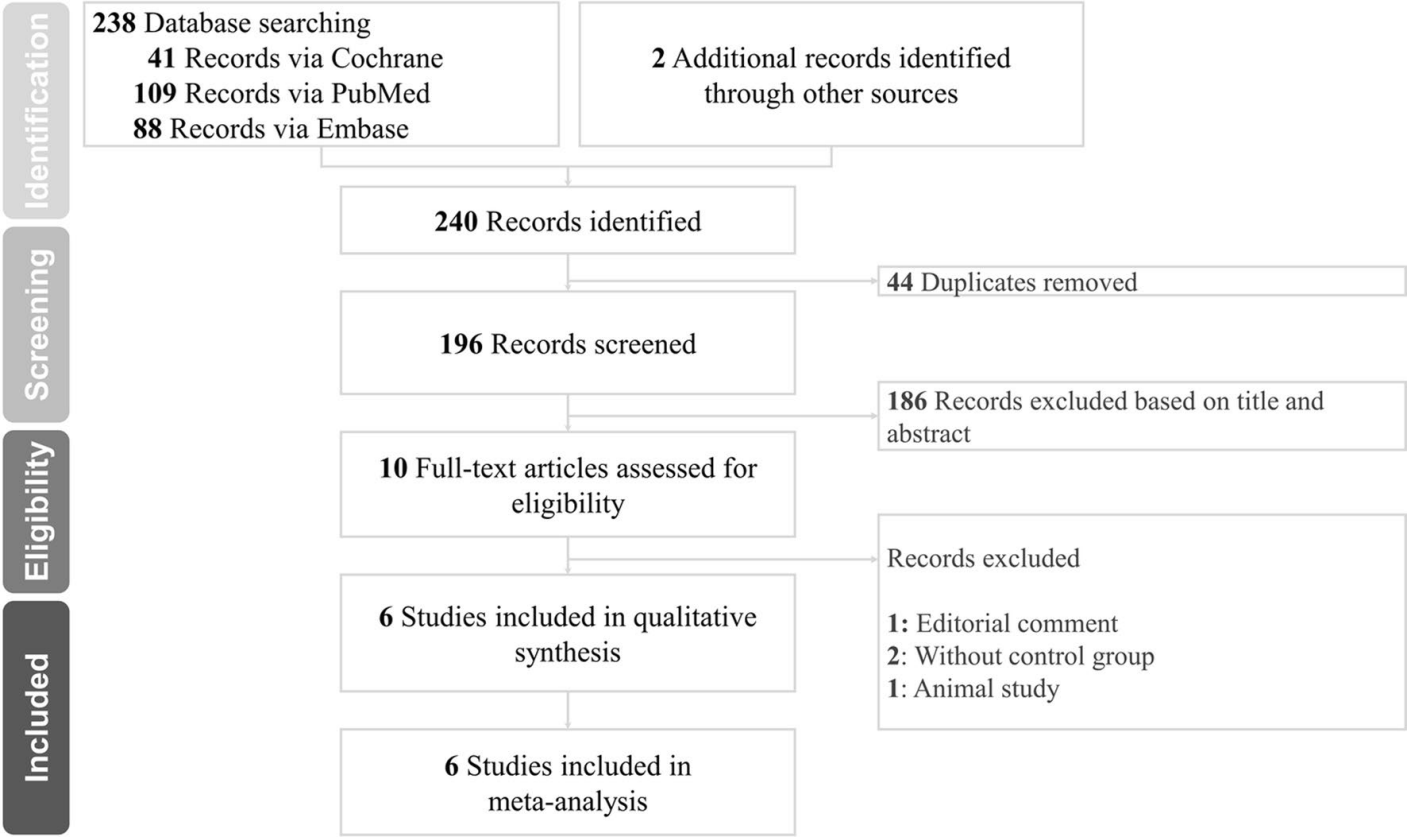
Flow diagram of the literature search

A total of 240 articles were identified in the initial step of the systematic literature search. After removing 44 duplicated and 186 articles by screening the titles and abstracts, the full-text review was performed for the remaining 10 potentially eligible studies. Finally, we included six articles in this review [12–17]. Figure 1 shows a flow diagram explaining the processes involved in identifying and including/excluding studies. The literature search strategy is summarized in Additional file 1: eTable 1.

### Demographics

Two randomized studies and four nonrandomized studies are presented in Table 1, which provides a general overview of patient demographics in the six articles. Among the six studies included, two types of ND were observed in the reported data. Radical ND referred to surgical clearance of all five lymph node (LN) groups (I–V), and selective ND consisted of the clearance of less than five LN groups of the ipsilateral neck. The pooled prevalence of the type of ND did not show a significant intergroup difference (*P* = 0.97). Additional file 1: eTable 2 and eTable 3 describe the results of the quality assessment. Two types of incisions, hidden and minimal, were used for END among the six studies. The hidden incisions, such as retroauricular or facelift incisions, were placed mainly behind the auricle and on the hairline to improve the cosmesis after ND. Minimal incisions, such as small submandibular or small suprasternal incisions, were used to perform LN dissection through a small cutaneous incision to reduce scarring after surgery.

**Table 1:** Basic characteristics of the included studies

Authors	Year	Country	Study Design	Age (Mean, yr)	Sex (M/F)	Follow-up	Incision type	Incision Site	Type of ND	Sample Size^*^	
						(Mean, m)				END	CND
Fan et al.	2014	China	RCT	51.7	21/23	34	Minimal	SM	Selective	23	21
Sannikorn et al.	2015	Thailand	Retrospective	53.3	47/23	NR	Hidden	RAFL	Selective	10	60
Fan et al.	2016	China	RCT	53.6	38/22	NR	Minimal	SM	Selective	31	29
Raj et al.	2016	India	Retrospective	NR	47/10	NR	Minimal	SS	Selective	36	21
Pawar et al.	2020	India	Retrospective	53.1	34/7	NR	Hidden	RAFL	Selective/Radical	21	20
Shah et al.	2020	India	Retrospective	49.4	66/6	24	Hidden	RAFL	Selective/Radical	32	48
										153	199

yr, year; m, month; M, male; F, female; ND, neck dissection; SM, submandibular; RAFL, retroauricular or facelift; SS, suprasternal; NR, not reported

*Number of sides of neck dissection

### Outcomes

#### LN yield

**Fig. 2:**
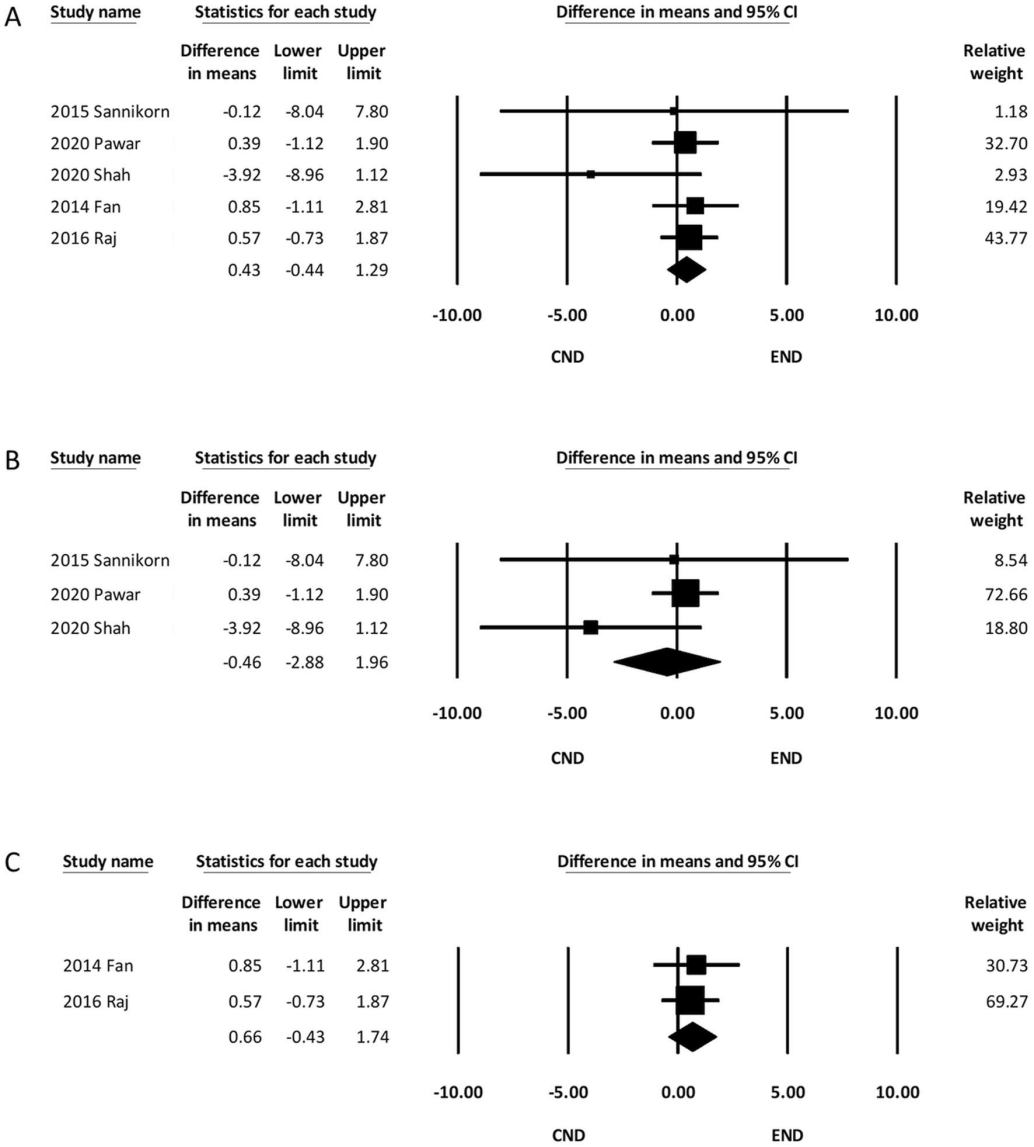
Forest plot of the LN yield. **A** Overall study group. **B** Studies using hidden incisions in END. **C** Studies using minimal incisions in END. CI, confidence interval; CND, conventional neck dissection; END, endoscope-assisted neck dissection

Five of the six included studies reported the number of LNs retrieved from ND [12–17]. The pooled analysis of the overall study group did not demonstrate a significant difference between the two groups regarding the number of LNs yielded (MD, 0.43; 95% confidence interval [CI], − 0.44 to 1.29) (Fig. 2A). Meta-analysis of the three studies using hidden incisions revealed a similar number of LNs yielded between the two ND groups (MD, − 0.46; 95% CI − 2.88 to 1.96) (Fig. 2B) [13, 16, 17]. Meta-analysis of the two studies using minimal incisions also showed no intergroup differences (MD, 0.66; 95% CI − 0.43 to 1.74) (Fig. 2C) [12, 15].

#### Operative time

**Fig. 3:**
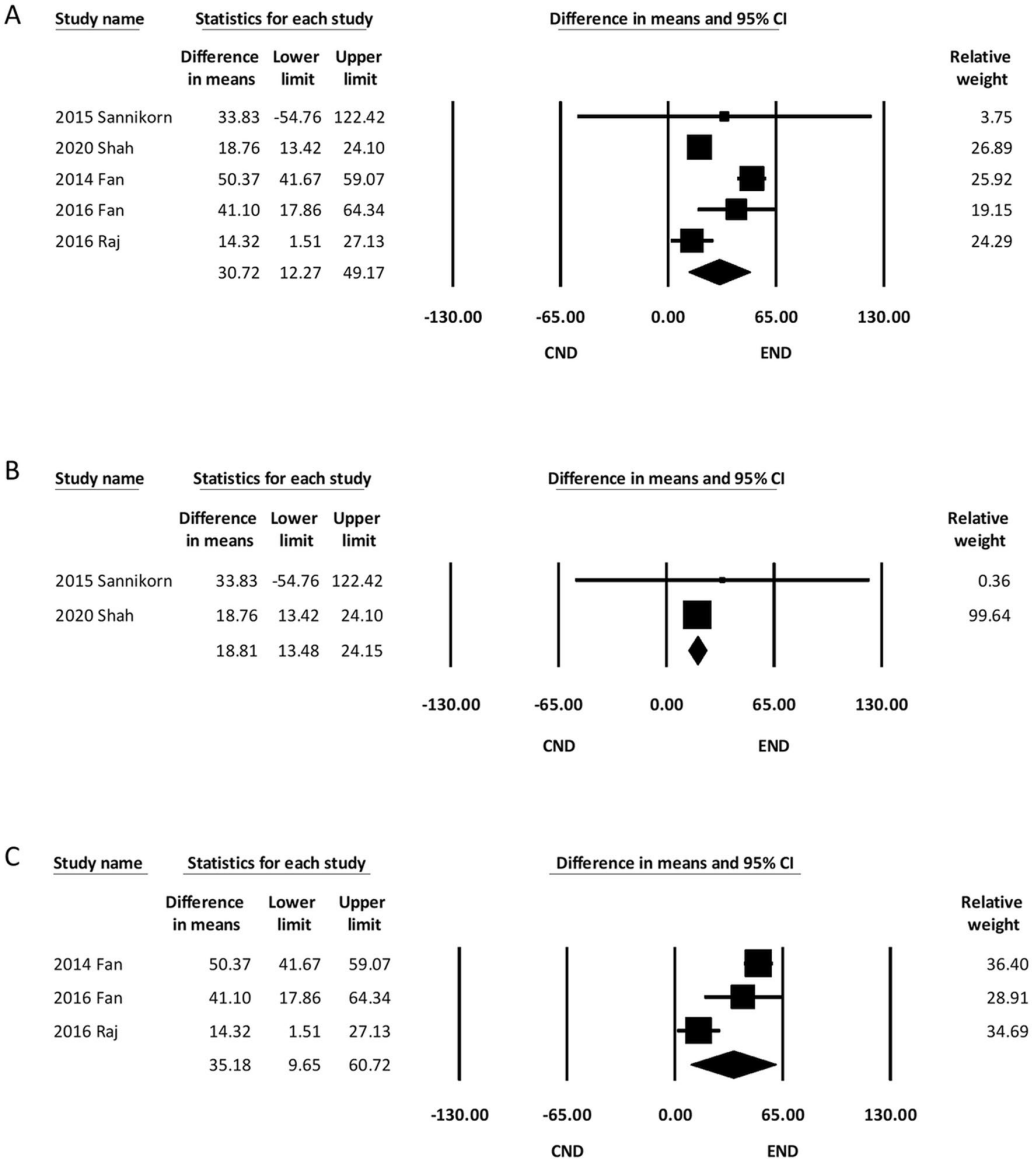
Forest plot of the operative time. **A** Overall study group. **B** Studies using hidden incisions in END. **C** Studies using minimal incisions in END. CI, confidence interval; CND, conventional neck dissection; END, endoscope-assisted neck dissection

Five of the six studies recorded the operative time needed to complete ND [12–15, 17]. The pooled results of the overall study groups showed that the operative time was longer in the END group than the CND group (MD, 30.72; 95% CI 12.27 to 49.17). An approximately 30-minute difference in the operative time was observed (Fig. 3A). Meta-analysis of the two hidden-incision studies showed that the operative time was longer in the END group (MD, 18.81; 95% CI 13.48 to 24.15) (Fig. 3B) [13, 17]. Meta-analysis of the three minimal-incision studies also revealed that the operative time was longer in the END group (MD, 35.18; 95% CI 9.65 to 60.72) (Fig. 3C) [12, 14, 15]**.** Further subgroup analysis was not performed due to the limited number of eligible studies.

#### Intraoperative blood loss

**Fig. 4:**
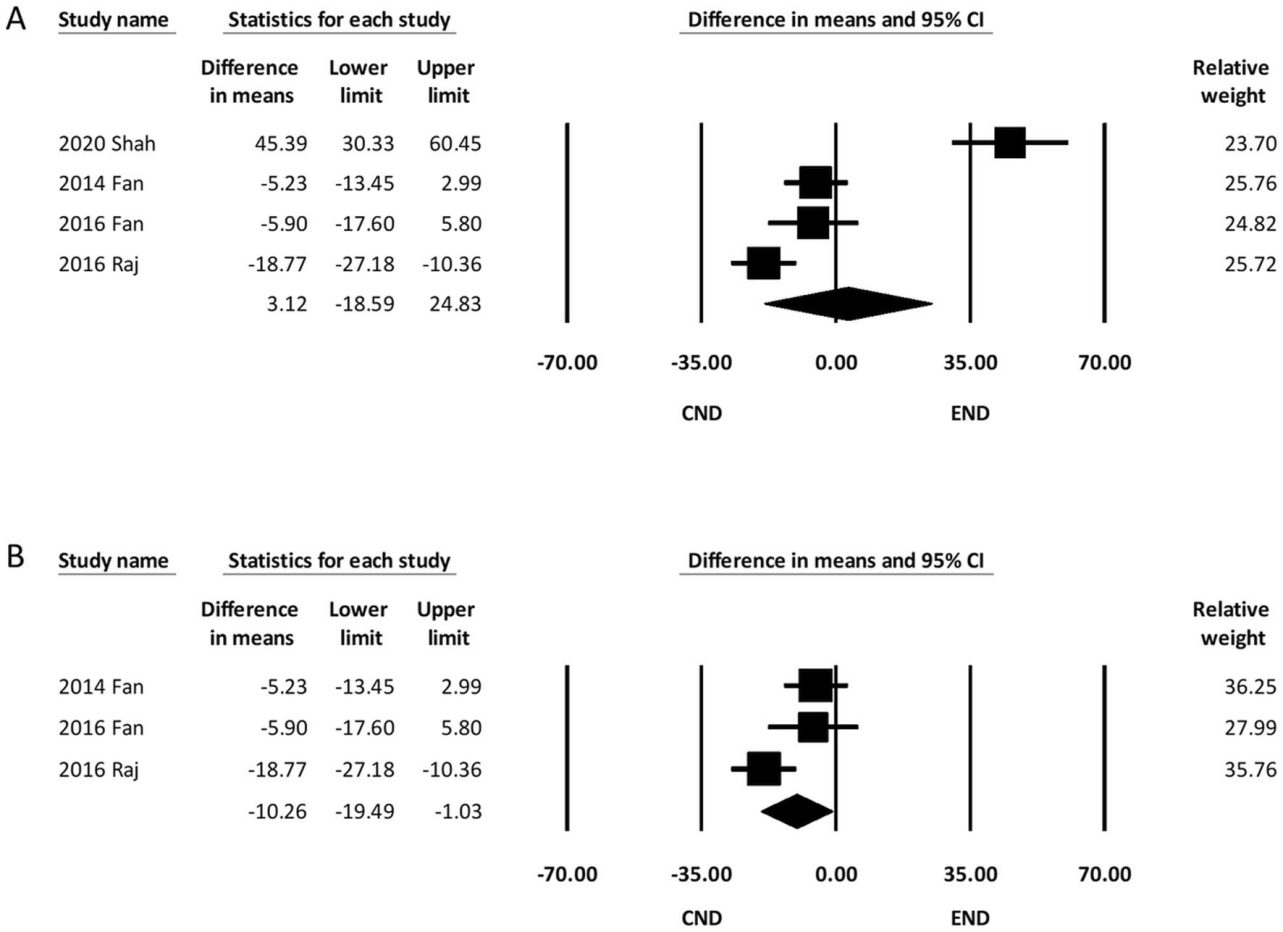
Forest plot of intraoperative blood loss. **A** Overall study group. **B** Studies using minimal incisions in END. CI, confidence interval; CND, conventional neck dissection; END, endoscope-assisted neck dissection

Four of the six studies reported intraoperative blood loss in ND [12, 14, 15, 17]. The pooled analysis showed comparable amounts of intraoperative blood loss between the two groups (MD, 3.12; 95% CI − 18.59 to 24.83) (Fig. 4A). Meta-analysis of hidden-incision studies could not be performed due to limited study numbers. A meta-analysis including three studies using minimal incisions demonstrated that intraoperative blood loss was lower in the END group (MD, − 10.26; 95% CI − 19.49 to − 1.03) (Fig. 4B) [12, 14, 15]. Subgroup analysis according to types of incision showed that the subgroup difference between hidden- and minimal-incision studies was significant, suggesting that it was a potential source of heterogeneity (*P* for subgroup difference < 0.001).

#### Length of hospital stay

**Fig. 5:**
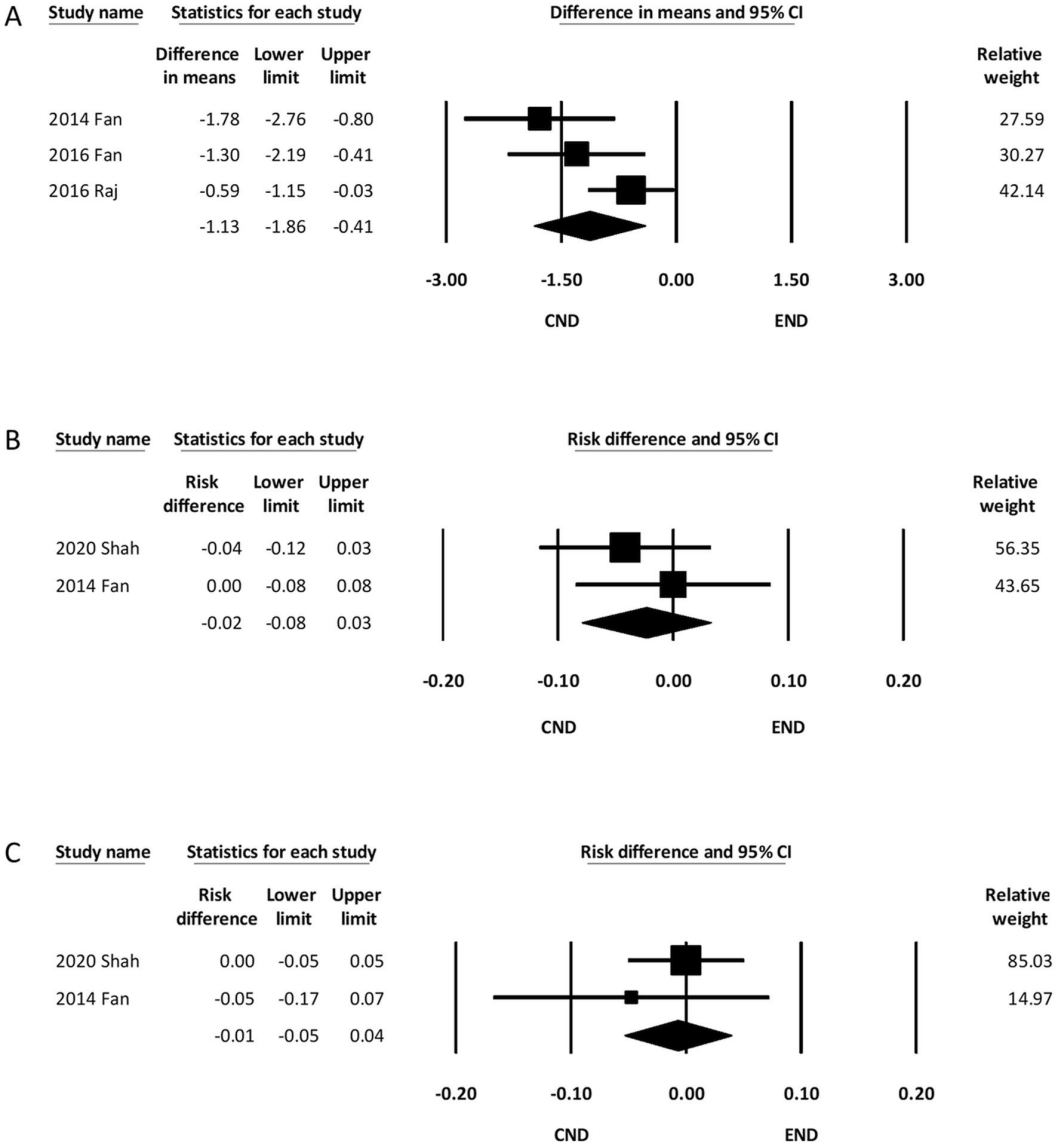
Forest plot of the length of hospital stay and recurrence. **A** Forest plot of the length of hospital stay. **B** Forest plot of local recurrence in the ipsilateral neck. **C** Forest plot of regional recurrence in the ipsilateral neck. CI, confidence interval; CND, conventional neck dissection; END, endoscope-assisted neck dissection

The three minimal-incision studies recorded the length of hospital stay after ND [12, 14, 15]. The pooled results showed that the length of hospital stay was shorter in the END group (MD, − 1.13; 95% CI − 1.86 to − 0.41) (Fig. 5A).

#### Ipsilateral nodal recurrence

Two of the studies recorded the local and regional nodal recurrence of the ipsilateral neck in the follow-up period [12, 17]. The pooled results showed that the incidence of local nodal recurrence was similar between the two groups (RD, − 0.02; 95% CI − 0.08 to 0.03) (Fig. 5B). The pooled results also demonstrated no significant intergroup difference regarding the incidence of regional nodal recurrence (RD, − 0.01; 95% CI − 0.05 to 0.04) (Fig. 5C).

#### Postoperative marginal mandibular nerve injury

**Fig. 6:**
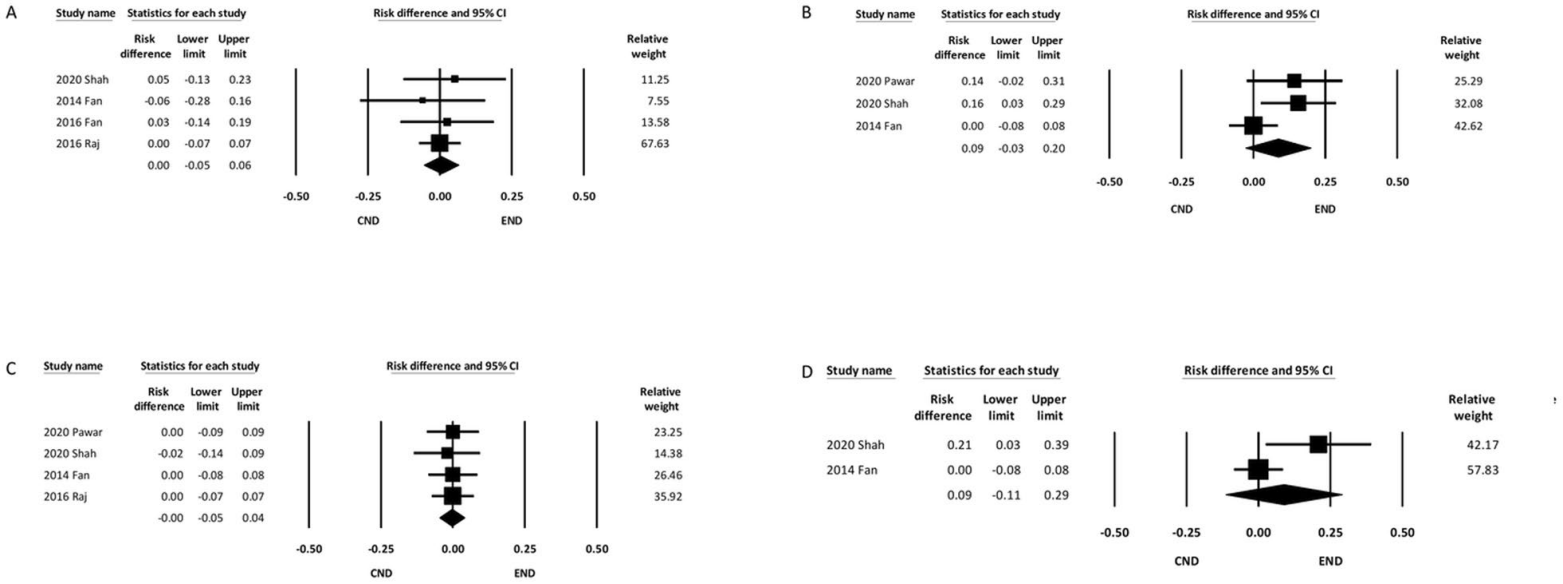
Forest plot of postoperative complications. **A** Marginal mandibular nerve injury. **B** Skin-edge necrosis. **C** Hematoma or bleeding. **D** Seroma. CI, confidence interval; CND, conventional neck dissection; END, endoscope-assisted neck dissection

The incidence of marginal mandibular nerve injury was reported by four of the included studies [12, 14, 15, 17]. Temporary injury was observed in two studies [12, 14]. Two other studies did not specify whether the injury was temporary or permanent [15, 17]. The data, regardless of the type of nerve injury, were pooled for analysis, and the results demonstrated comparable incidence between the two groups (RD, 0.00; 95% CI − 0.05 to 0.06) (Fig. 6A). Pooled analysis of the two studies reporting temporary marginal mandibular nerve injury also revealed a similar incidence between the two groups (Additional file 1: eFigure 1).

#### Postoperative skin edge necrosis

Three of the six studies reported the incidence of skin edge necrosis [12, 16, 17]. The pooled analysis did not demonstrate a significant intergroup difference (RD, 0.09; 95% CI − 0.03 to 0.20) (Fig. 6B).

#### Postoperative hematoma or bleeding

Four of the six studies reported the incidence of hematoma or bleeding after ND [12, 15–17]. The pooled results demonstrated a comparable incidence of postoperative hematoma or bleeding (RD, − 0.00; 95% CI − 0.05 to 0.04) (Fig. 6C).

#### Postoperative seroma

Two of the six studies reported the incidence of seroma formation after ND [12, 17]. The pooled results showed that the incidence was similar between the two groups (RD, 0.09; 95% CI − 0.11 to 0.29) (Fig. 6D).

### Publication bias

Additional file 1: eTable 4 shows the funnel plots as well as the results of Egger's and heterogeneity tests. No evident publication bias was noted based on Egger's intercept test.

## Discussion

The present meta-analysis was performed to report the differences between END and CND in the existing English literature. According to our results, the LN yield was similar between the two ND techniques. The intraoperative blood loss, locoregional recurrence rate, and postoperative complications also showed no intergroup differences. In addition, the END group demonstrated a shorter hospital stay and a significantly longer operative time than the CND group. To the best of our knowledge, this is the first comparison of these two ND techniques in a systematic review.

Cervical LN metastasis is recognized as one of the most important prognostic factors in oral cancers [18]. Therefore, ND plays a fundamental role in the surgical management of oral cancers, and the extent of dissection is planned according to the disease severity as well as the therapeutic program [19]. Conventionally, ND is performed through a long transcervical incision to remove LNs of the neck. However, an obvious cutaneous scar is inevitable after ND, causing cosmetic concerns in some patients. Incision within the potential radiation field is another issue for postoperative treatment [20]. Surgery via remote access or minimal incision was thus studied. A previous meta-analysis of robotic ND reported the features of this modern technique [20]. However, the availability of robotic systems, the training of personnel, the learning curve, and the overall cost for patients present challenges in making robotic ND a routine practice in most hospitals [21, 22]. In contrast, the adequate cost-effectiveness and easy accessibility of the endoscope-assisted approach make it an alternative treatment modality [21]. One recent review article, focusing on endoscope-assisted lateral ND in thyroid cancer patients, also demonstrated a similar concept [23]. The authors showed that not only was endoscope-assisted lateral ND feasible but that it also provided more cosmetically pleasing results than the conventional open approach and a comparable LN yield and complication rate. On the other hand, the present study discusses the differences between the two ND techniques in oral cancer patients and provides information regarding locoregional recurrence according to currently available data.

The therapeutic goal of cervical lymphadenectomy is to completely remove LNs that are at potential risk [24]. The approaches used to perform ND, whether conventional or novel, should be able to provide (theoretically, at least) equivalent outcomes regarding this goal. The number of LNs retrieved from ND, that is, the LN yield, has been reported to be positively associated with survival outcome in several studies [25, 26]. In addition, a meta-analysis has also suggested that LN yield could be a valuable indicator for the quality of ND [27]. The LN yield between the END and CND groups was comparable according to our pooled results. Subgroup analysis of END using hidden incision or minimal incision showed that both techniques offer a similar LN yield as the CND group. These findings suggested that the use of END did not decrease or increase the LN yield compared with that from CND. Long-term oncological outcomes were not reported in the majority of the studies included, which precludes further survival analysis. However, information regarding local and regional recurrence in the ipsilateral neck was reported by two studies. The pooled analysis from these two studies indicated that the rate of recurrence did not differ significantly between the two groups. Future studies on the topic of long-term and more detailed oncological outcomes are necessary for comprehensive analysis.

According to our meta-analysis, the operative time was significantly longer in the END group. This result is intuitive because the subplatysmal dissection and creation of a surgical field from either the hidden or minimal incision is more time consuming than the CND procedure. Among the five studies reporting operative time, four demonstrated a significant increase in the operative time in the END group. An intergroup difference of approximately 30 min was observed in our pooled analysis. Similar results were observed in a previous review comparing ND between robotic and conventional techniques [5]. The authors reported that robotic ND takes approximately 70 min longer than CND [5]. The further prolonged operative duration of robotic ND may be due to the time needed for docking and repositioning of the robotic system. The difference in the operative time needed for robotic ND or END, despite not being revealed by direct comparison, may be another issue for surgeons when considering alternative approaches in cervical lymphadenectomy.

The operative blood loss in the overall study group was comparable between the two ND techniques. Pooled analysis of studies using minimal incisions showed that the operative blood loss was lower in the END group. Possible explanations may involve the use of endoscopic dissection to minimize vessel injuries and the compression effect of CO_2_ insufflation reported by some authors [12, 15]. The incidences of postoperative complications were also calculated in the present study. The pooled results demonstrated that the incidences of postoperative marginal mandibular nerve injury, skin edge necrosis, hematoma/bleeding and seroma were all similar between the two groups. During the surgical steps of ND, careful dissection remained the fundamental factor regardless of the approach. These results suggested that the use of END did not increase or decrease the occurrence of common complications. The pooled result revealed a shorter of hospital stay in the END group and, more specifically, in studies using minimal incisions. The minimized wound may contribute to the recovery process; similar findings have also been reported in thyroidectomy using minimal incisions [28]. The cosmesis of the neck after surgery was reported to be superior in five of the included studies [12, 14–17]. However, only one study recorded the satisfaction score based on a numerical scale, which prevented further statistical analyses [12]. The use of END by hidden or minimal incision may offer better cosmesis after surgery; however, more studies with validated satisfaction assessments are needed to confirm this result.

Several limitations should be acknowledged in this review. First, only six studies were included in the analysis. More studies are required for a more comprehensive evaluation. Second, although two articles were randomized trials, four retrospectively designed studies were also included due to data availability. Third, heterogeneity between studies was found in some parameters, suggesting that these results need to be interpreted with caution.

## Conclusions

Compared with CND, the END procedure demonstrated comparable results regarding LN yield, intraoperative blood loss, complication rate, and locoregional recurrence. In addition, the operative time required for ND was significantly longer in the END group, and the hospital stay was shorter. The use of END may offer potential benefits compared with CND regarding cosmetic outcomes. The present study reveals that the oncological outcomes of END do not seem to be inferior to those of CND; however, further research is still required due to the limited number of studies included in our meta-analysis.

## Supplementary Information

**Additional file 1: eTable 1.** Literature Searches and Keywords. **eTable 2.** Newcastle-Ottawa Scale Quality Assessment of Included Non-randomized Studies. **eTable 3.** Risk of Bias Assessment of Included Randomized Studies. **eTable 4.** Funnel plots. **eFigure 1.** Funnel plots of temporary marginal mandibular nerve injury.

## Data Availability

The datasets used and/or analyzed during the current study are available from the corresponding author on reasonable request.

## Abbreviations

LN: Lymph node
ND: Neck dissection
END: Endoscope-assisted neck dissection
CND: Conventional neck dissection
MDs: Mean differences
RDs: Risk differences
