# Supplementary material for: Endoscope-assisted versus conventional neck dissection in patients with oral cancer: a systematic review and meta-analysis
Source: J Otolaryngol Head Neck Surg. 2022 May 11;51:20. doi: 10.1186/s40463-022-00567-9 (PMC9097363; doi:10.1186/s40463-022-00567-9)
Supplement: Supplementary file 1 — Additional file 1: eTable 1. Literature Searches and Keywords. eTable 2. Newcastle-Ottawa Scale Quality Assessment of Included Non-randomized Studies. eTable 3. Risk of Bias Assessment of Included Randomized Studies. eTable 4. Funnel plots. eFigure 1. Funnel plots of temporary marginal mandibular nerve injury. [file 40463_2022_567_MOESM1_ESM.doc]

**eTable 1.** Literature Searches and Keywords

| - Medline   ((((((("endoscopy"[All Fields] OR "endoscope"[All Fields] OR "endoscopic"[All Fields] OR "endoscope-assisted"[All Fields] OR "video"[All Fields]) AND ("neck dissection"[All Fields] OR "neck lymphadenectomy"[All Fields] OR "cervical lymphadenectomy"[All Fields] OR "neck lymph node dissection"[All Fields] OR "cervical lymph node dissection"[All Fields])) NOT "review"[Publication Type]) NOT "meta-analysis"[Publication Type]) AND "English"[Language]) NOT ("larynx"[Title/Abstract] OR "laryngeal"[All Fields])) NOT (("thyroid"[Title/Abstract] OR "thyroidectomy")[Title/Abstract])) NOT (("robot"[Title/Abstract] OR "robotic"[Title/Abstract] OR "laser"[Title/Abstract] OR "tonsil"[Title/Abstract] OR "tonsillar"[Title/Abstract] OR "esophagus"[Title/Abstract] OR "esophageal"[Title/Abstract] OR "sentinel")[Title/Abstract]) |
| --- |
| - EMBASE   ('endoscopy':ti,ab,kw OR 'endoscope':ti,ab,kw OR 'endoscopic':ti,ab,kw OR 'endoscope-assisted':ti,ab,kw OR 'video':ti,ab,kw) AND ('neck dissection':ti,ab,kw OR 'neck lymphadenectomy':ti,ab,kw OR 'cervical lymphadenectomy':ti,ab,kw OR 'neck lymph node dissection':ti,ab,kw OR 'cervical lymph node dissection':ti,ab,kw) AND english:la NOT 'case report':it NOT ('review'/exp OR review) NOT ('meta analysis'/exp OR 'meta analysis') NOT ('larynx'/exp OR 'larynx' OR 'laryngeal') NOT ('thyroid'/exp OR 'thyroid' OR 'thyroidectomy'/exp OR 'thyroidectomy') NOT ('robot'/exp OR 'robot' OR 'robotic' OR 'laser'/exp OR 'laser' OR 'tonsil'/exp OR 'tonsil' OR 'tonsillar' OR 'esophagus'/exp OR 'esophagus' OR 'esophageal' OR 'sentinel'/exp OR 'sentinel') |
| - Cochrane library   ("endoscopy" OR "endoscope" OR "endoscopic" OR "endoscope-assisted" OR "video") in Title Abstract Keyword AND ("neck dissection" OR "neck lymphadenectomy" OR "cervical lymphadenectomy" OR "neck lymph node dissection" OR "cervical lymph node dissection") in Title Abstract Keyword NOT case report in Publication Type NOT review in Publication Type NOT meta-analysis in Publication Type - (Word variations have been searched) |

eTable 2: Newcastle-Ottawa Scale Quality Assessment of Included Non-randomized Studies

| **Study ID** | **Selection** | | | | **Comparability*** | **Outcome** | | | **Total (**⋆**)** |
| --- | --- | --- | --- | --- | --- | --- | --- | --- | --- |
|  | Representativeness of the Exposed Cohort | Selection of the Non-Exposed Cohort | Ascertainment of Exposure | Demonstration that Outcome of Interest was not Present at Start of Study | Comparability of Cohorts on the Basis of the Design or Analysis | Assessment of Outcome | Follow-up was  Long Enough  for Outcomes  to Occur | Adequacy of  Follow-up of  Cohorts |  |
| Sannikorn 2015 | ⋆ | ⋆ | ⋆ | ⋆ | ⋆ | ⋆ |  | ⋆ | 7 |
| Raj 2016 | ⋆ | ⋆ | ⋆ | ⋆ | ⋆ | ⋆ |  | ⋆ | 7 |
| Pawar 2020 | ⋆ | ⋆ | ⋆ | ⋆ | ⋆⋆ | ⋆ |  | ⋆ | 8 |
| Shah 2020 | ⋆ | ⋆ | ⋆ | ⋆ | ⋆⋆ | ⋆ | ⋆ | ⋆ | 9 |

eTable 3: Risk of Bias Assessment of Included Randomized Studies

| **Study ID** | Random sequence generation | Allocation concealment | Blinding of participants and personnel | Blinding of outcome assessment | Incomplete outcome data | Selective reporting | Other biases |
| --- | --- | --- | --- | --- | --- | --- | --- |
| Fan 2014 | Unclear | Unclear | High | High | Low | Low | Unclear |
| Fan 2016 | Unclear | Unclear | High | High | Low | Low | Unclear |

**eTable 4:** Funnel plots

| **Parameter** | ***I^2^*** | **Egger’s Test** | **Funnel Plot** |
| --- | --- | --- | --- |
| Lymph nodes yield (overall) | 0.000 | 0.243 | 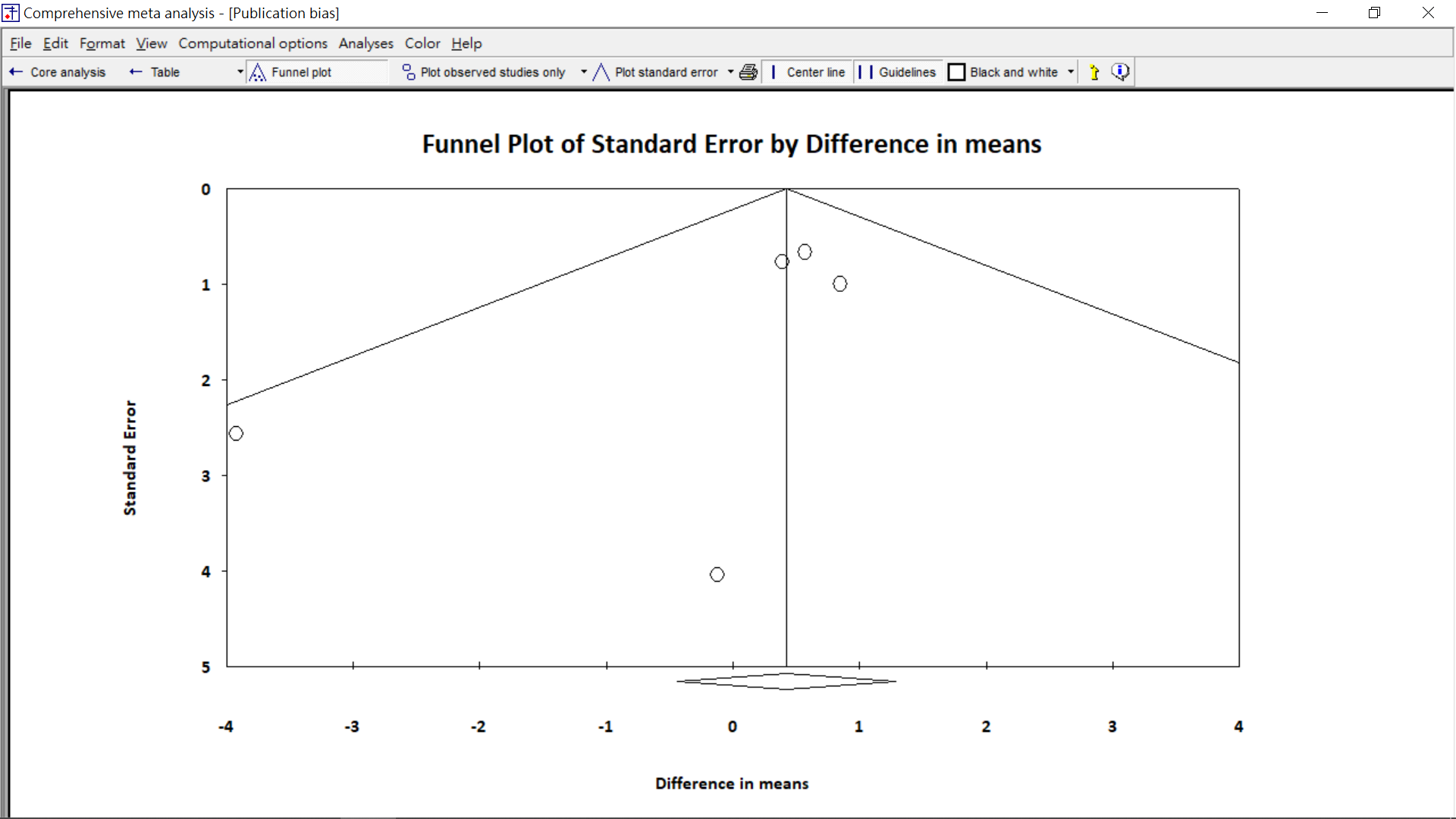 |
| Lymph nodes yield (Hidden incision) | 22.529 | 0.545 | 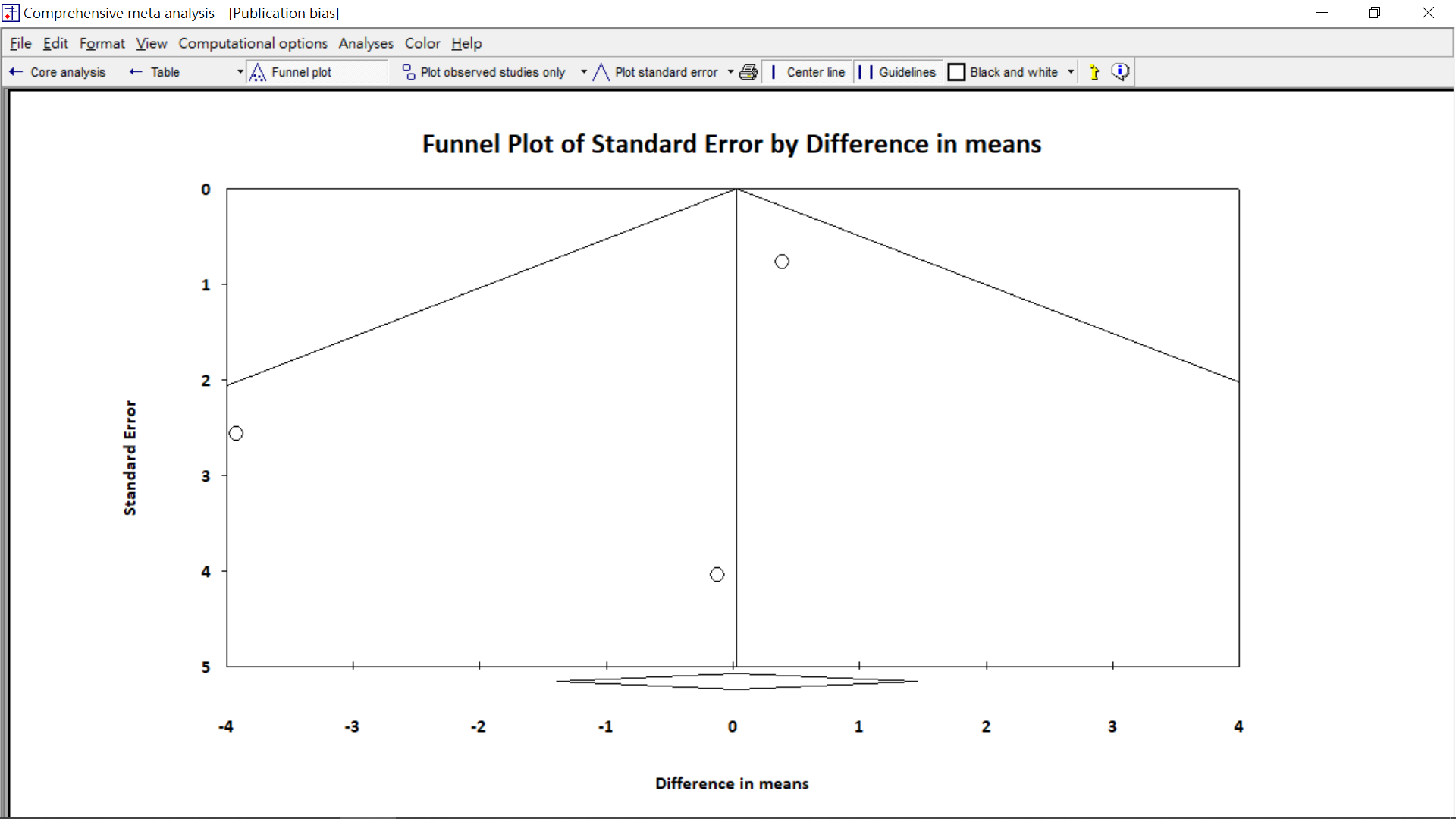 |
| Lymph nodes yield (Minimal incision) | 0.000 | X | X |
| Operative time (overall) | 90.472 | 0.682 | 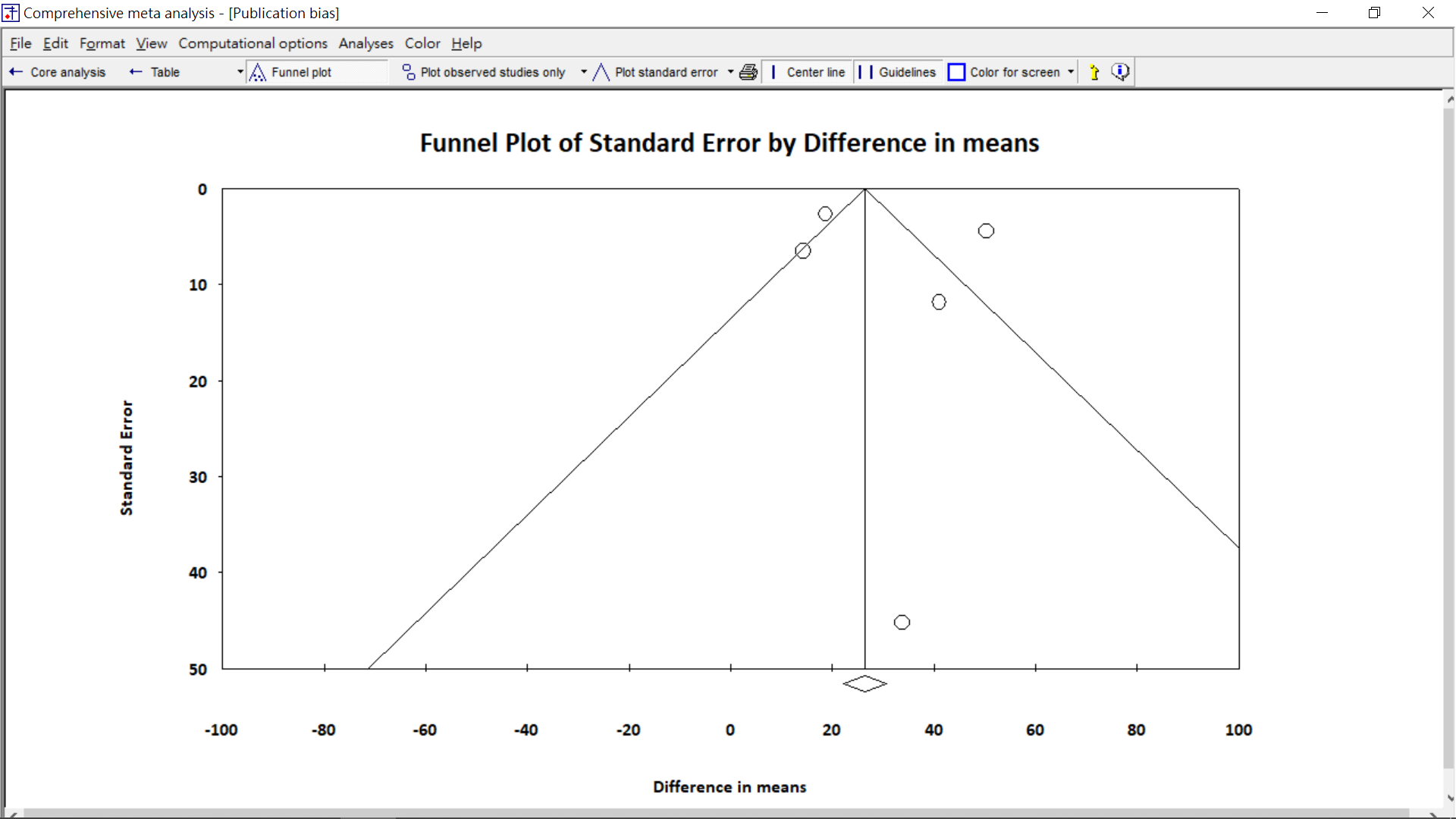 |
| Operative time (Hidden incision) | 0.000 | X | X |
| Operative time (Minimal incision) | 90.408 | 0.735 | 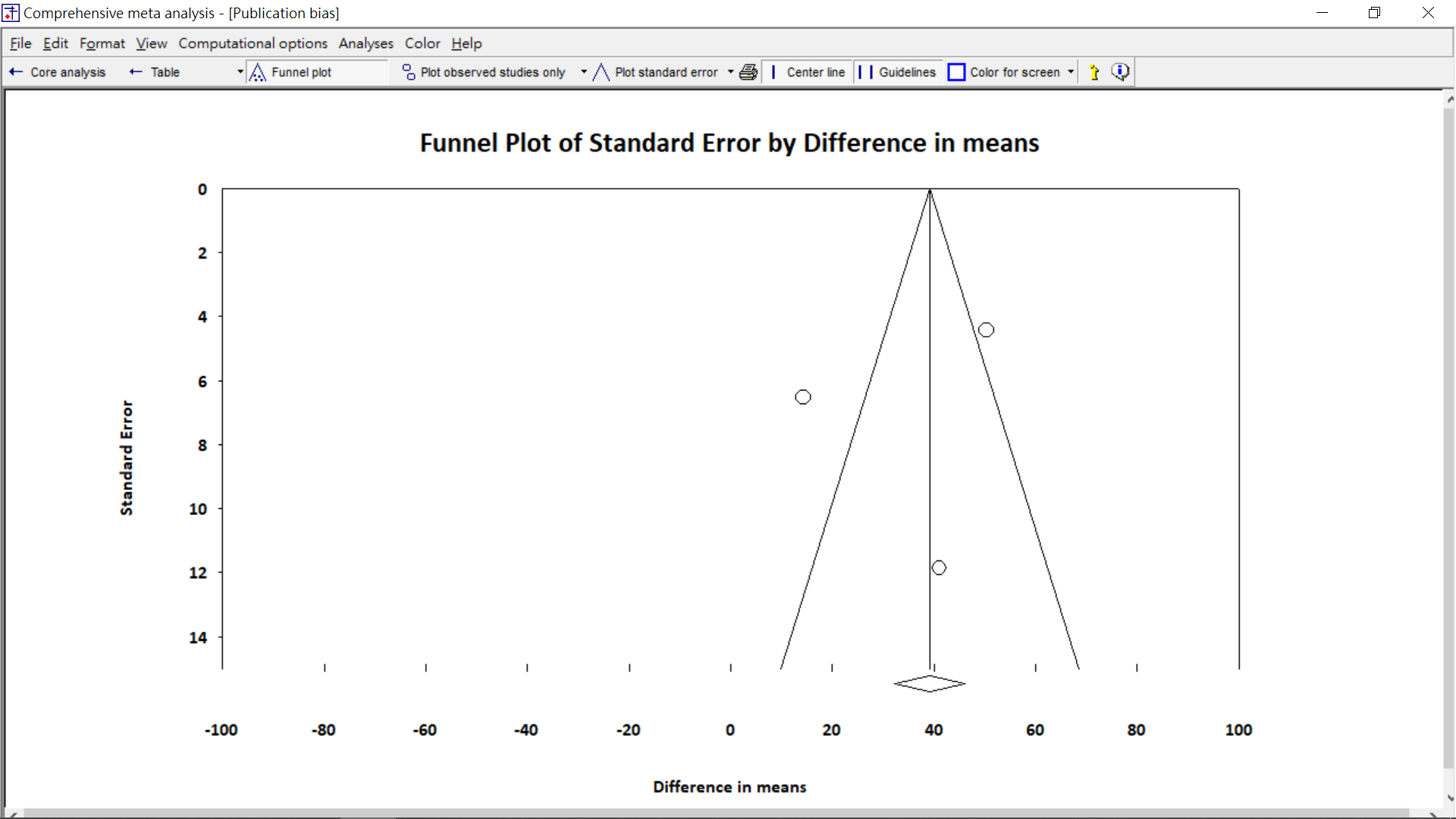 |
| Intraoperative blood loss (overall) | 94.371 | 0.163 | 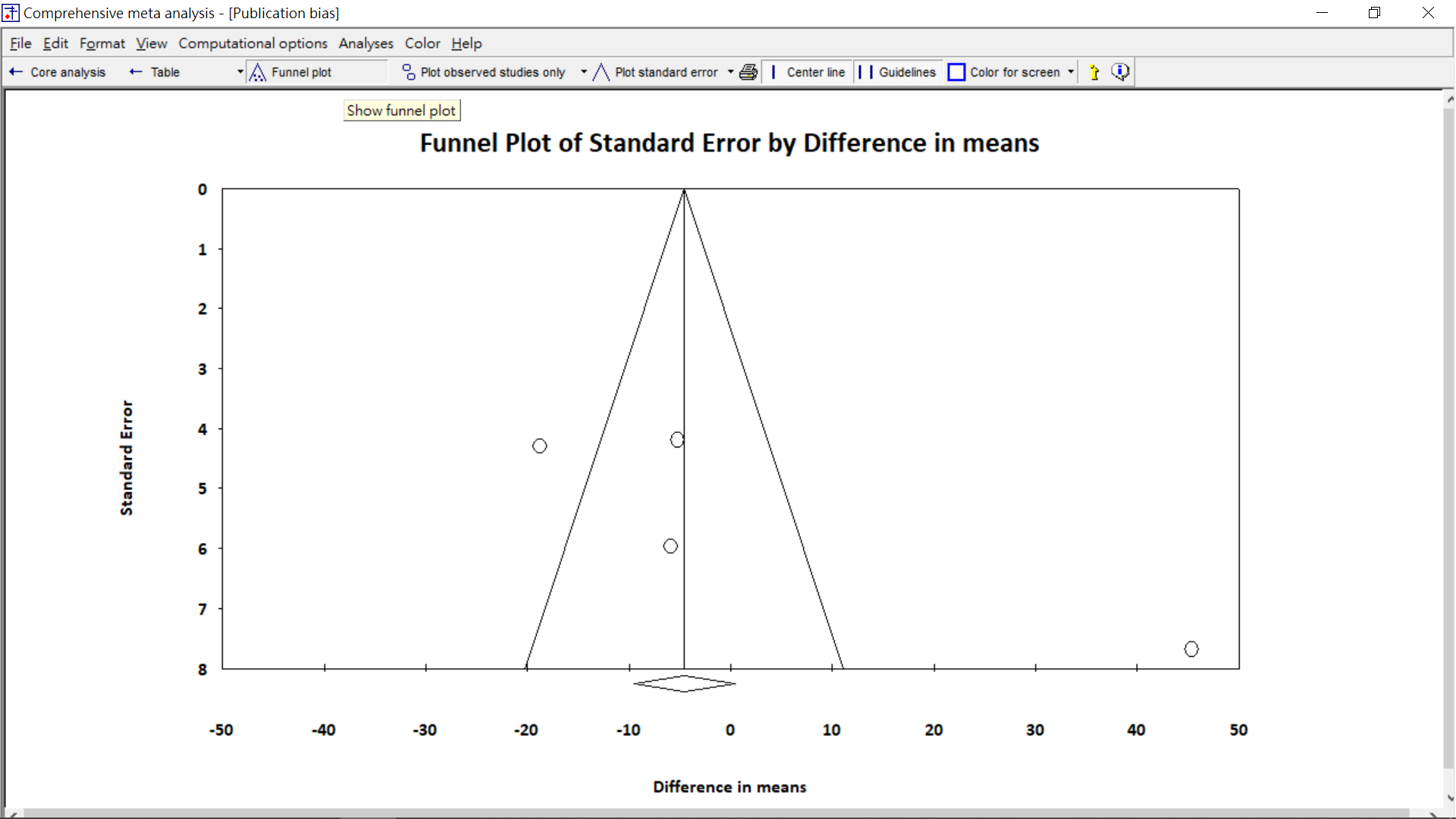 |
| Intraoperative blood loss (Minimal incision) | 62.987 | 0.801 | 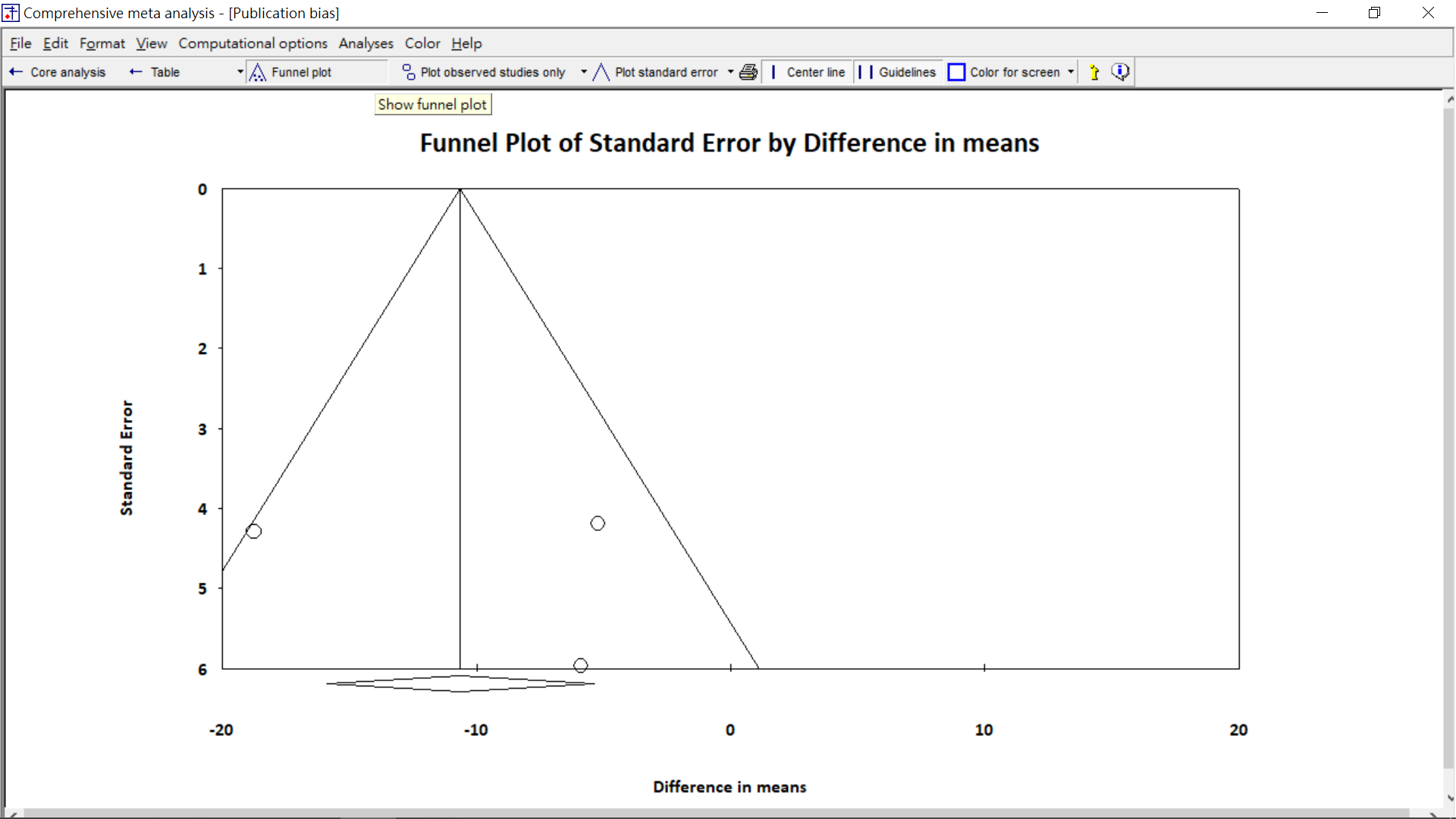 |
| Length of hospital stay | 59.279 | 0.109 | 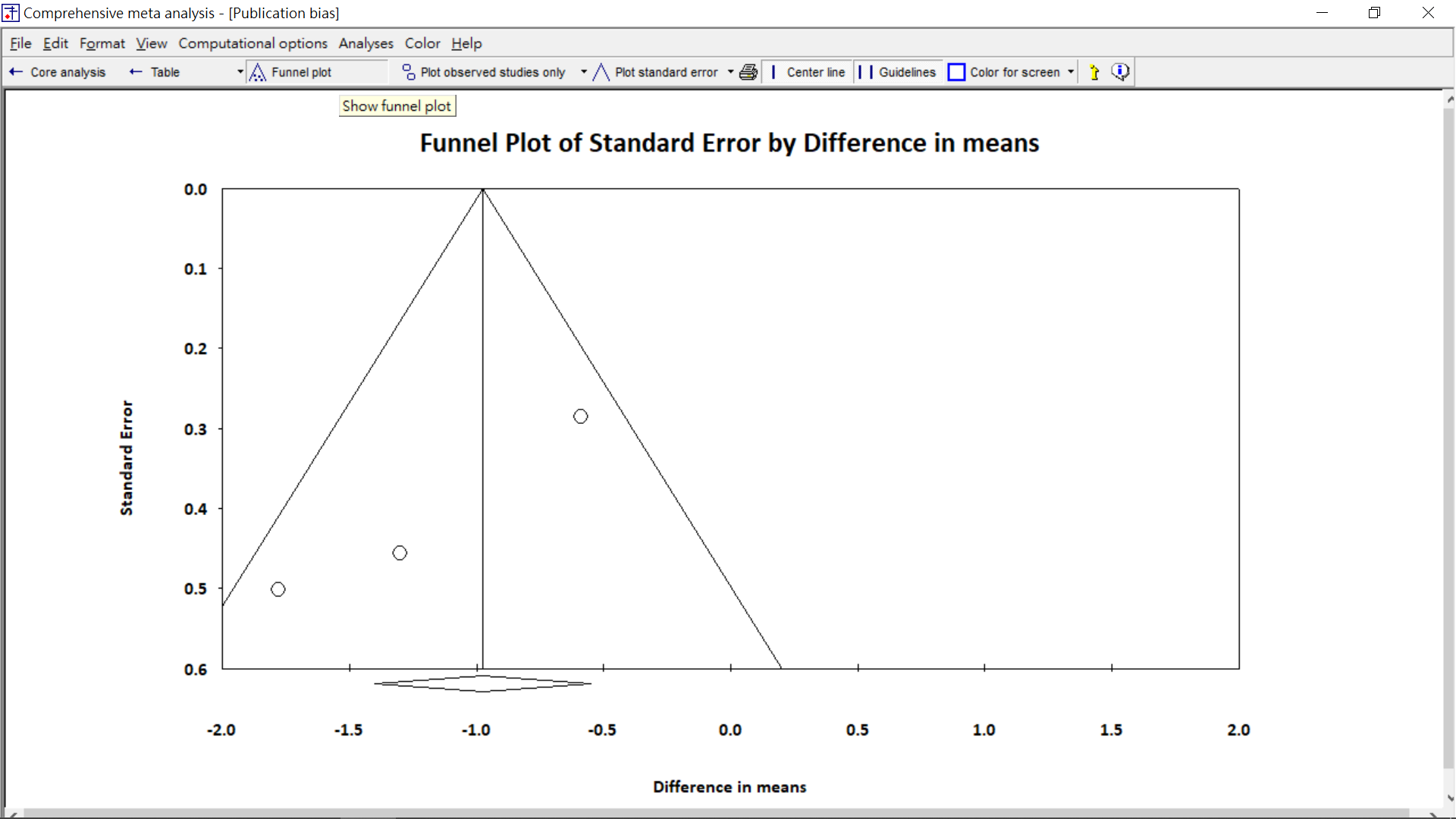 |
| Ipsilateral local nodal recurrence | 0.000 | X | X |
| Ipsilateral regional nodal recurrence | 0.000 | X | X |
| Marginal mandibular nerve injury | 0.000 | 0.920 | 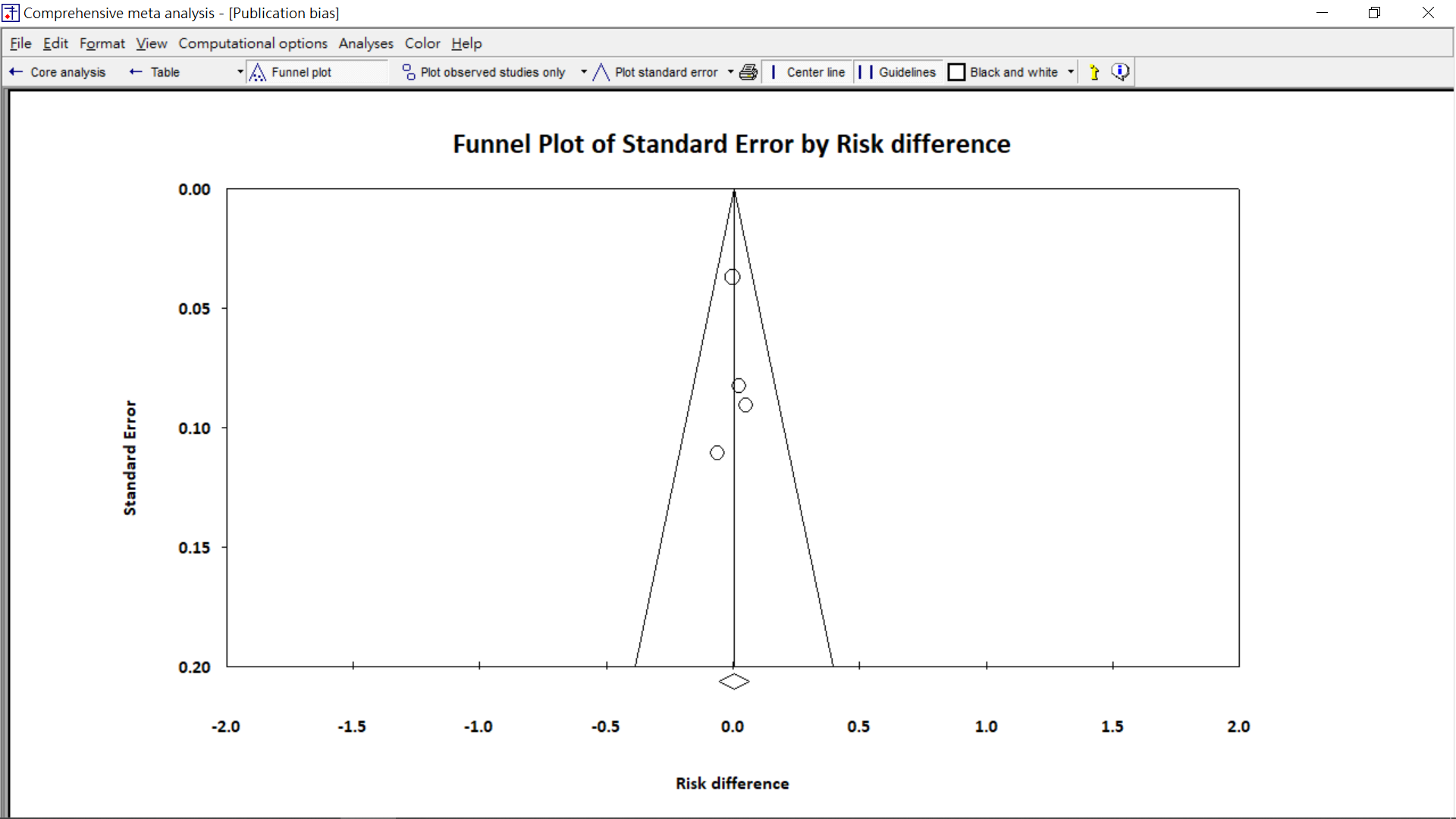 |
| Skin edge necrosis | 59.906 | 0.267 | 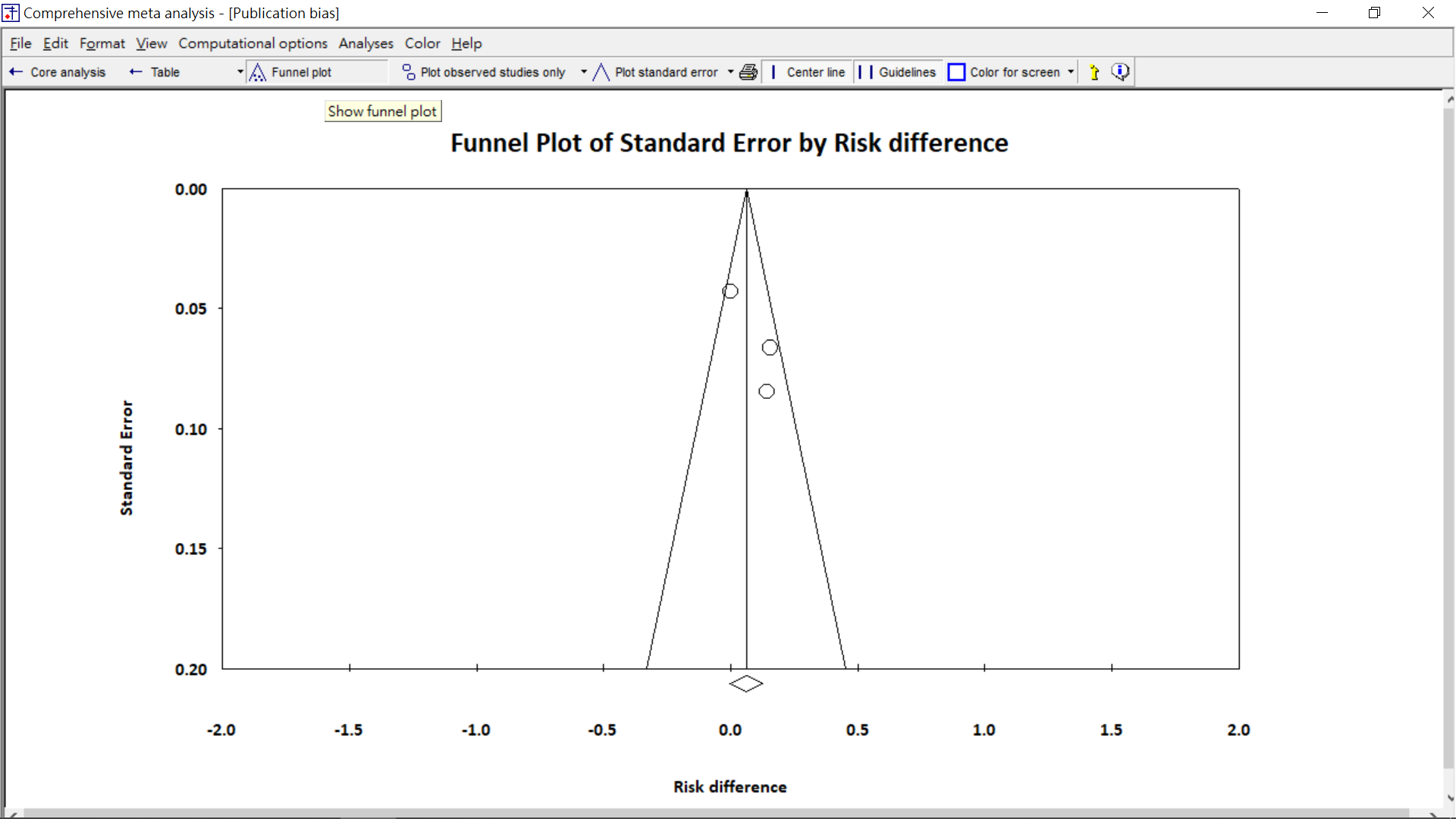 |
| Postoperative hematoma/bleeding | 0.000 | 0.139 | 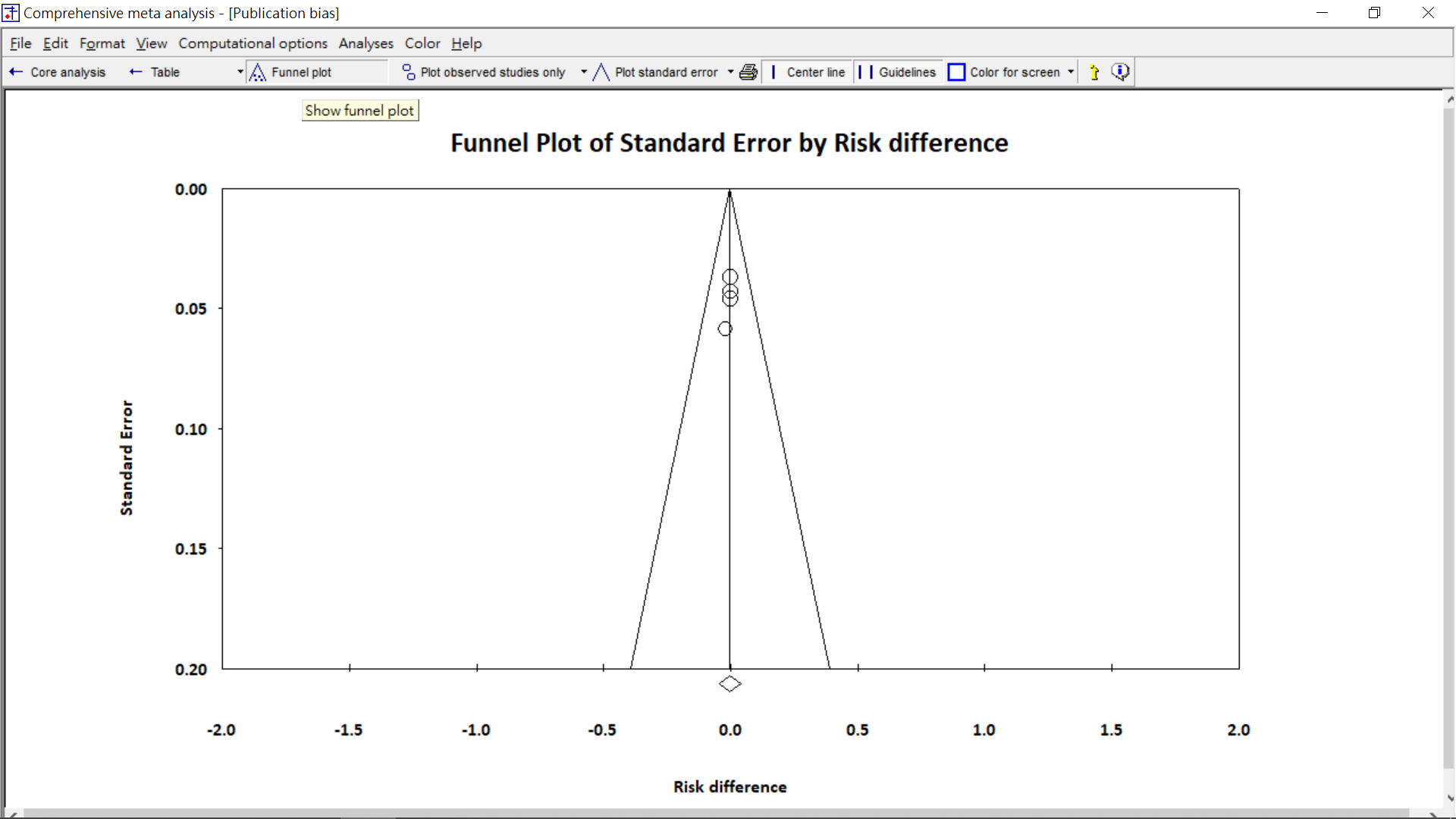 |
| Postoperative seroma | 75.768 | X | X |

**eFigure 1:** Funnel plots of temporary marginal mandibular nerve injury
